# Glibenclamide Nanocrystal-Loaded Bioactive Polymeric Scaffolds for Skin Regeneration: In Vitro Characterization and Preclinical Evaluation

**DOI:** 10.3390/pharmaceutics13091469

**Published:** 2021-09-14

**Authors:** Julie R. Youssef, Nabila A. Boraie, Heba F. Ibrahim, Fatma A. Ismail, Riham M. El-Moslemany

**Affiliations:** 1 Department of Pharmaceutics, Faculty of Pharmacy, Alexandria University, Alexandria 21523, Egypt; gs-julie.wadie@alexu.edu.eg (J.R.Y.); nabila.boraie@alexu.edu.eg (N.A.B.); fatma.ismail@alexu.edu.eg (F.A.I.); 2 Department of Histology and Cell Biology, Faculty of Medicine, Alexandria University, Alexandria 21523, Egypt; heba.fathy15@alexmed.edu.eg

**Keywords:** glibenclamide, nanocrystals, collagen, chitosan, bioactive, scaffold, wound healing

## Abstract

Skin restoration following full-thickness injury poses significant clinical challenges including inflammation and scarring. Medicated scaffolds formulated from natural bioactive polymers present an attractive platform for promoting wound healing. Glibenclamide was formulated in collagen/chitosan composite scaffolds to fulfill this aim. Glibenclamide was forged into nanocrystals with optimized colloidal properties (particle size of 352.2 nm, and polydispersity index of 0.29) using Kolliphor as a stabilizer to allow loading into the hydrophilic polymeric matrix. Scaffolds were prepared by the freeze drying method using different total polymer contents (3–6%) and collagen/chitosan ratios (0.25–2). A total polymer content of 3% at a collagen/chitosan ratio of 2:1 (SCGL3-2) was selected based on the results of in vitro characterization including the swelling index (1095.21), porosity (94.08%), mechanical strength, rate of degradation and in vitro drug release. SCGL3-2 was shown to be hemocompatible based on the results of protein binding, blood clotting and percentage hemolysis assays. In vitro cell culture studies on HSF cells demonstrated the biocompatibility of nanocrystals and SCGL3-2. In vivo studies on a rat model of a full-thickness wound presented rapid closure with enhanced histological and immunohistochemical parameters, revealing the success of the scaffold in reducing inflammation and promoting wound healing without scar formation. Hence, SCGL3-2 could be considered a potential dermal substitute for skin regeneration.

## 1. Introduction

The skin is the largest organ of the body, playing a pivotal role in sensation, thermoregulation and protection from environmental hazards [1]. However, wounds disrupt this protective barrier, predisposing the body to infections and allowing for protein and water loss [2]. Histologically, wound healing is described to have three overlapping stages: inflammation, proliferation and remodeling [3]. Following injury, platelets, formed fibrin clots and injured nerves provide signals inducing inflammation, vasodilation and increased capillary permeability to facilitate the migration of inflammatory cells [4]. The proliferation phase is characterized by the influx of fibroblasts, extracellular matrix (ECM) deposition, formation of new blood vessels and re-epithelialization [5]. The last stage, remodeling, outlined by both wound contraction and collagen remodeling, aims to achieve the maximum tensile strength through reorganization, degradation and re-synthesis of the extracellular matrix.

For optimum wound healing, an ideal wound dressing should retain a moist environment, augment epidermal migration, reduce inflammation and promote angiogenesis and connective tissue synthesis. Additionally, it should allow for gas exchange between the wounded tissue and the surrounding environment [2]. Although synthetic materials have excellent mechanical properties and are easily processed, they have a major drawback, which is their low biocompatibility [6]. Hence, modern dressings are formulated from natural polymers such as alginate, collagen, chitosan, elastin and hyaluronic acid, mimicking the structural and biological characteristics of the skin’s extracellular matrix (ECM) [2]. These polymers are known for their biocompatibility, biodegradability and non-toxic nature, in addition to their bioactivity, which aid in wound healing [7].

Collagen is the major constituent of the extracellular matrix (ECM) and acts as a depot for cell adhesion and proliferation [8]. Its metabolites are small bioactive peptides possessing wound healing ability [9]. Collagen is widely considered as an appropriate choice for a variety of restorative applications due to its unique biological properties, relative abundance in living tissue [10], excellent biocompatibility, well-balanced biodegradability and weak antigenicity [11]. Despite its advantages, collagen has weak mechanical strength and a fast biodegradation rate, which are critical problems limiting its application [8]. To overcome these problems, blending with other natural polymers is the solution to modify the biodegradation rate and optimize the mechanical properties [12]. Chitosan, a natural biocompatible and biodegradable polysaccharide with a well-established safety profile certified by the US FDA [13], is widely used in wound dressings [14]. It possesses hemostatic properties in addition to antimicrobial, anticancer and anti-inflammatory actions [15]. The combination of chitosan and collagen provided improved scaffold mechanical properties and controlled degradation [12]. Moreover, non-medicated collagen/chitosan scaffolds were shown to promote wound healing [10,16]. Collagen composite scaffolds for the purpose of wound healing were also prepared using other natural and synthetic polymers such as polycaprolactone [2], hyaluronic acid/gelatin [17] and fibrin [18].

Medicated wound dressings are intended to accelerate the healing process. Topical drugs purposed to accomplish this function include antibiotics, antioxidants, anti-inflammatories and drugs enhancing tissue regeneration [19]. Along these lines of conventional topical drugs, repurposed drugs are also applied for healing. The use of repurposed drugs offers the advantage of a less risky and more rapid return on investment, with lower average associated costs once failures have been accounted for. Examples of these repurposed drugs are calcium channel blockers [20], statins [21], phenytoin [22] and antidiabetic drugs [23]. Recent studies were performed on the wound healing potential of different classes of antidiabetic medications including sulfonylureas, thiazolidinediones, dipeptidyl peptidase-4 inhibitors and metformin [24]. The sulphonyl urea group including glibenclamide, glimepiride and gliclazide [25] was specifically studied for its healing potential as, in addition to insulin regulation, fat and carbohydrate metabolism is also controlled, providing a better healing environment and stimulating keratinocyte cellular proliferation and formation of granulation tissue [2]. Moreover, they were shown to possess a high anti-inflammatory action [26], especially glibenclamide, as it contains both sulfonyl and benzamido groups [27]. Glibenclamide was shown to decrease the secretion and mRNA expression of pro-inflammatory cytokines such as IL-1β and IL-18 that are predominantly produced by macrophages in the wounded region [27]. Additionally, it was shown to be a potential inhibitor of collagenases, offering additional advantages in controlling MMP activation and collagen degradation [28]. Improvement in the healing cascade was reported upon the combination of these repurposed antidiabetic drugs with natural bioactive polymers for their pivotal role in healing. Cam et al. [26] showed accelerated wound healing by topical application of a combination of oral antidiabetic agents loaded in chitosan and gelatin nanofibrous scaffolds. Additionally, loading of glibenclamide and metformin into bacterial cellulose/gelatin fibrous scaffolds offered a high potential for diabetic wound healing with a high bioavailability and fewer systemic side effects [29].

The problem facing the incorporation of most of the repurposed drugs into hydrophilic bioactive polymeric scaffolds is their high lipophilicity. Interestingly, drug formulations as nanocrystals could allow for better dispersion [30], due to their small size and high surface/volume ratio [31]. Nanocrystals developed for topical application were shown to enhance esthetics and improve drug wettability, dissolution, stability and adhesion [32]. Moreover, nanocrystals showed enhancement in the release profile of drugs [30], also providing localized drug delivery to the wound site [31].

In this context, the current study aimed to formulate glibenclamide (GL) as a repurposed drug model for wound healing. GL nanocrystals were prepared, evaluated and loaded into collagen/chitosan bioactive composite scaffolds. The developed scaffolds were characterized in vitro for pharmaceutical attributes including swelling, degradability and mechanical strength and in vivo for their ability to promote wound healing.

## 2. Materials and Methods

### 2.1. Materials

Glibenclamide (GL), collagen (CO, molecular weight ≈ 300 KDa) and chitosan (CS, high molecular weight, 310–375 KDa, degree of acetylation is 95%) were a kind gift from Pharco Pharmaceuticals Co., Alexandria, Eygpt. Glutaraldehyde (GA) was purchased from Adwic, El-Nasr Pharmaceutical Co., Cairo, Egypt. Cetyltrimethylammonium bromide (CTAB), polyvinyl alcohol (PVA, molecular weight, 98 KDa) and Kolliphor HS15 (K-HS15) were purchased from Sigma-Aldrich (St. Louis, MO, USA). Bovine serum albumin was obtained from Biowest, Riverside, MO, USA. Lysozyme was from Bio Basic, Markham, ON, Canada. Sodium hydroxide, sodium lauryl sulphate (SLS), polyvinyl pyrrolidone K30 (PVP, molecular weight 40 KDa), polyethylene glycol 4000 and calcium chloride were from El-Gomhoureya chemicals, Alexandria, Egypt. Dimethyl sulfoxide (DMSO), absolute ethanol, methanol and acetic acid were of analytical grade.

### 2.2. Preparation of Glibenclamide Nanocrystals

Glibenclamide nanocrystals (NC-GL) were prepared using the previously reported bottom-up nanoprecipitation method with some modifications [33,34]. PVP K30, PEG 4000, PVA, SLS, poloxamer 188 and Kolliphor HS15 (K-HS15) were tested for NC-GL stabilization. Formulation codes, compositions and conditions are listed in Table 1. Briefly, GL 2.5% *w*/*v* in DMSO was poured into the aqueous stabilizer’s solution under continuous magnetic stirring at 800 rpm in an ice bath for 30 s for immediate precipitation of NC-GL in the antisolvent (stabilizer solution). The obtained dispersion was then probe sonicated at 40 kHz (Sonoplus HD 3100; BANDELIN, Berlin, Germany) in an ice bath for 5–15 min. The obtained nano-suspensions were centrifuged (Laboratory centrifuge 3K-30; Sigma, Osterode, Germany) at 16,000 rpm for 30 min at 4 °C to separate nanocrystal pellets.

### 2.3. Preparation of Collagen/Chitosan Scaffold

For the preparation of composite scaffolds, CO and CS were separately dissolved in 1% aqueous acetic acid at room temperature. The scaffolds were prepared by mixing CO and CS solutions in different ratios (0.25–2) with a total polymer content of 3 and 6% [1]. The CO/CS ratio and total polymer content used throughout the study were selected based on preliminary investigations (results not shown). Scaffold codes and compositions are shown in Table 2. The CO/CS mixtures were magnetically stirred for 1 h, and pH was adjusted to 5.5. Gluteraldehyde (GA, 0.1% of the total polymer weight) was then added for crosslinking, and stirring continued for 5–15 min based on the total polymer content. Following stirring, the casting solutions were poured into circular molds of 12 mm diameter and 4 mm depth. The molds were frozen at −80 °C for 24 h followed by lyophilization (Lyoquest; Telstar, Terrassa, Spain) to obtain the scaffolds.

GL-loaded scaffolds were prepared by adding the selected optimized NC-GL formulation to the CO/CS mixture before crosslinking with adequate stirring to ensure a homogenous distribution before pouring into the molds to yield a final GL concentration of 2 mg/scaffold.

### 2.4. In Vitro Characterization

#### 2.4.1. Colloidal Properties

The mean particle size (PS) and polydispersity index (PDI) of the NC-GL formulations were analyzed by dynamic light scattering (DLS) using a Malvern Zetasizer^®^ at a fixed angle (173°) at 25 °C using a 4 mW He-Ne laser at 633 nm (Zetasizer^®^ Nano ZS series DTS 1060, Malvern Instruments S.A, Worcestershire, UK). Zeta potential (ZP) was determined at 25 °C using a cell voltage of 150 V and 5 mA current. Prior to analysis, the dispersions were suitably diluted with filtered deionized water. Measurements were performed in triplicate.

#### 2.4.2. Scanning Electron Microscopy (SEM)

The microstructure of the GL coarse powder, freeze-dried NC-GL and CO/CS scaffolds either blank or loaded with NC-GL was observed under SEM (100 CX; JEOL, Tokyo, Japan). GL, NC-GL and scaffolds cut into small blocks were fixed on metal supports and sputter coated with gold palladium under an argon atmosphere prior to examination.

#### 2.4.3. Solid State Properties

Solid state properties of GL, NC-GL, CO, CS and selected blank and loaded scaffolds were investigated by the following methods.

##### Fourier Transmission Infrared Spectroscopy (FTIR)

FTIR spectra were obtained by a diamond ATR spectrophotometer (model: Cary 630, Agilent Biotechnology, Penang, Malaysia). Samples (2–5 mg) were placed in a diamond ATR sample holder and scanned over the wave number range of 4000–500 cm^−1^.

##### Differential Scanning Calorimetry (DSC)

The thermal behavior was examined by DSC (LINSEIS STA PT1000 TG-DTA/DSC, Bruker, Berlin, Germany). Samples were sealed in an aluminum crimp cell and heated at a constant 10 °C/min rate from 30 to 500 °C under nitrogen atmosphere purged at a flow rate of 60 mL/min. A control empty pan was subjected to the same conditions and used as reference.

##### Powder X-ray Diffraction (PXRD)

The crystalline nature was evaluated using X-ray diffraction (XRD, Bruker D2-Phaser; Madison, WI, USA). A copper radiation source was used (wavelength 1.54184 Å) at a voltage of 30 kV and a current of 10 mA. The diffraction pattern was obtained in a step scan model in the 2θ range 10° to 40°, with a step size of 0.02°.

##### Thermal Gravimetric Analysis

The thermal decomposition kinetics were investigated by thermogravimetric analysis using an STA 449F3 Jupiter (Netzsch, Selb, Germany) thermogravimetric analyzer. The weight of each sample was 18 mg, the heating rate was 50 mL/min and the temperature region was 35–500 °C. Then, the thermal gravimetric curves of the samples were plotted.

#### 2.4.4. Porosity and Pore Size

The liquid displacement method was used to measure porosity using absolute ethanol as the displacing liquid [35]. First, scaffold dimensions were estimated using a Vernier caliper to calculate the volume, the dry weight was measured and then the scaffold was immersed in a graduated cylinder enclosing a known volume of absolute ethanol (5 mL) and soaked for 24 h at room temperature, allowing ethanol to penetrate the pores; then, the final weight of the wet scaffold was noted. Percentage porosity was calculated using the following equation:

$$\%\;\mathrm{Porosity}=\frac{\left(W_{f}-W_{i}\right)/\rho_{ethanol}\;}{V}\times 100$$

where W_i_ and W_f_ are the weights of the scaffold before and after immersion in absolute ethanol, respectively, V is the scaffold volume and ρ_ethanol_ is the density of ethanol.

Moreover, the average pore size was calculated from at least 20 measurements on SEM images using image analysis software (Fiji version 1.52p; National Institutes of Health, Bethesda, MD, USA).

#### 2.4.5. Swelling Behavior

Swelling behavior of scaffolds was assessed by measuring the water uptake potential. Scaffolds were immersed in 5 mL phosphate buffer saline (PBS) pH 7.4 at 37 °C. The scaffolds were taken out at predetermined time intervals (3, 6 and 24 h) and weighed after blotting excess water against filter papers [36]. Percentage swelling was calculated using the equation

$$\mathrm{Swelling}\;\%=\frac{W_{w}-W_{d}}{W_{d}}\times 100$$

where W_d_ is the dry weight, and W_w_ is the weight after immersion in PBS.

#### 2.4.6. Water Holding Capacity

The swollen scaffold was kept at 37 °C in an incubator for 12 h. At regular time intervals, the scaffolds were removed for weighing. Water holding capacity (**R**) was calculated according to the following formula [37]:

$$R=\frac{W_{t}}{W_{o}}\times 100$$

where W_o_ is the weight of the swollen scaffold, and W_t_ is the weight of the scaffold at time intervals.

#### 2.4.7. In Vitro Biodegradation

In vitro biodegradability of the scaffolds was studied by lysozyme digestion. Scaffolds (*n* = 3) were immersed in 2 mL lysozyme solution in PBS pH 7.4 and incubated at 37 °C for 30 days. The lysozyme solution was refreshed every 2 days to maintain constant enzyme activity [38]. To examine the scaffold degradation rate, the lysozyme solution was removed at indicated time points, and samples were washed with sterile deionized water, dried and weighed. Percentage degradation (%) was determined as follows [39]:

$$\%\;\mathrm{Degradation}=\frac{Wo-Wt}{Wo}\times 100$$

where W_o_ is the initial weight, and W_t_ is the weight of the scaffold at time t.

#### 2.4.8. Mechanical Properties

The mechanical behavior of scaffolds was studied using a texture analyzer (CT3; Brookfield, MA, USA) fitted with a 10 N load cell. Freeze-dried circular scaffolds of diameter 12 mm both dry and prewetted with saline for 4 h were compressed at a test speed of 15 mm/min. The ultimate compressive strength was recorded, and stress–strain curves were generated during compression [40]. Young’s compressive modulus was derived from the regression of the stress–strain curves’ linear region. Assessments were conducted in triplicates.

#### 2.4.9. Drug Release Study

##### In Vitro Dissolution Study of Nanocrystals

In vitro dissolution of NC-GL was performed on the selected lyophilized formulation; NC-GL7 compared to GL powder using the dialysis bag technique. Dialysis bags (Visking^®^, MWCO 12,000–14,000; SERVA, Heidelberg, Germany) were soaked in distilled water for 24 h prior to the experiment. Accurate amounts equivalent to 2 mg GL dispersed in 1 mL phosphate buffer pH 7.4 were filled in the dialysis bags which were then sealed and suspended in 50 mL phosphate buffer pH 7.4 containing CTAB 0.1% *w*/*v* to maintain sink conditions. The release medium was selected based on GL solubility study (data not shown). Samples were kept in a shaking water bath (Wisebath^®^, London, UK) at 37 °C at 100 rpm. At predetermined time intervals, 2.5 mL aliquots were withdrawn and replaced with fresh dissolution medium. Samples were analyzed spectrophotometrically (Cary 60 UV-Vis Spectrophotometer, Agilent, Santa Clara, CA, USA) at λ_max_ 300 nm. Measurements were conducted in triplicate.

##### In Vitro Glibenclamide Release from Scaffolds

GL release from scaffolds was investigated by enclosing the scaffolds in a nylon mesh and separately immersing them in the developed release medium (phosphate buffer pH 7.4 containing CTAB 0.1% *w*/*v*) simulating sink conditions in a shaking water bath at 100 rpm at 37 °C. Serial sampling over 7 days with replacement was conducted. Samples were filtered using 0.22 µm syringe filters and analyzed spectrophotometrically at λ_max_ 300 nm. Measurements were conducted in triplicate.

The release kinetics were determined by comparing the release profiles of scaffolds by fitting to different mathematical kinetic models and choosing the best fit by regression analysis of the plot. This was conducted using an Excel add-in, DDsolver, for modeling and comparison of drug release profiles [41].

### 2.5. In Vitro Hemocompatibility

#### 2.5.1. Protein Adsorption

Bovine serum albumin (BSA) adsorption was inspected using the batch contact method to understand the scaffold interaction with blood protein [42,43]. BSA was dissolved in PBS pH 7.4 at a concentration of 2 mg/mL. Scaffolds were swollen in PBS pH 7.4 for 2 h and then weighed after blotting on filter paper to remove surface liquid. Subsequently, the swollen scaffolds were soaked in 20 mL of the BSA solution at room temperature and shaken continuously for 30 min [43]. Then, the optical absorbance of BSA solution was recorded by UV spectrophotometry at 280 nm, and the concentration was obtained based on the BSA standard curve. The amount of BSA adsorbed on samples was calculated using the following equation:

$$Adsorbed\;BSA\;\left(mg/g\right)=\frac{C_{o}-C_{a}}{w}\times V$$

where C_o_ and C_a_ are the BSA concentration before and after protein adsorption on samples mg/mL), w is the weight of the swollen sample (g) and V is the volume of the BSA solution (mL).

#### 2.5.2. Whole Blood Clotting

The anticoagulant properties of the studied scaffolds were evaluated by blood clotting study. Blood was collected in sodium citrate tubes from the human ulnar vein of a healthy volunteer using a sterile syringe. Citrate/blood mixture (200 µL) was poured on each scaffold and then 20 µL of 0.2 M CaCl_2_ was added to each sample to initiate the clotting cascade [11]. Samples were incubated at 37 °C for 15 min, after which 1 mL distilled water was added slowly along the sides of the plate without disturbing the clot to hemolyze the RBCs that were not trapped in the clot. The resulting hemolysate was centrifuged at 6000 rpm for 10 min, and the supernatant was analyzed spectrophotometrically by measuring optical density (OD) at λ_max_ 545 nm. The control group was set as directly adding 200 µL of citrated blood to 1 mL of distilled water and incubated similarly.

#### 2.5.3. In Vitro Hemolysis Assay

The hemolysis test was evaluated by an established method [44]. Briefly, 2 mL of fresh human blood was collected from a healthy adult volunteer in sodium citrate as an anticoagulant and centrifuged at 2500 rpm for 10 min. Red blood cells (RBCs) were separated, washed three times with saline and diluted with normal saline in the ratio 4:5 by volume. Scaffolds were incubated with 10 mL physiological saline in centrifugal tubes at 37 °C for 30 min. Next, 200 μL diluted RBCs was added to the tubes and further incubated for 60 min at 37 °C. Subsequently, all tubes were centrifuged for 10 min at 6000 rpm. Finally, the absorbance of the supernatant was measured spectrophotometrically at 545 nm. Positive and negative controls were prepared by incubating hematocrit with distilled water and normal saline, respectively, under similar conditions. The percentage hemolysis was calculated according to the following equation:

$$\%Hemolysis=\frac{(A_{s}-A_{n})\;}{\left(A_{p}-A_{n}\right)}\times 100$$

where A_s_, A_p_ and A_n_ represent the absorbance of the sample, positive control (distilled water) and negative control (normal saline), respectively.

### 2.6. In Vitro Biocompatibility

#### 2.6.1. Cell Culture

The human skin fibroblast (HSF) cell line was obtained from Nawah Scientific Inc. (Mokatam, Cairo, Egypt). Cells were maintained in DMEM media (Gibco, New York, NY, USA) supplemented with 100 mg/mL of streptomycin, 100 units/mL of penicillin and 10% of heat-inactivated fetal bovine serum (Gibco, NY, USA) in a humidified, 5% (*v*/*v*) CO_2_ atmosphere at 37 °C.

#### 2.6.2. Cell Viability Assay

The effect of NC-GL compared to GL on in vitro cell viability was assessed by sulforhodamine B (SRB) assay [45]. Aliquots of 100 μL cell suspension (5 × 10^3^ cells) were seeded in 96-well plates, incubated in complete media and allowed to adhere for 24 h. The medium was then replaced by fresh medium containing serial dilutions of either GL or NC-GL. After 72 h of drug exposure, cells were fixed by replacing media with 150 μL of 10% trichloroacetic acid (TCA) and incubated at 4 °C for 1 h. The TCA solution was removed, and the cells were washed 5 times with distilled water. Aliquots of 70 μL SRB solution (0.4% *w*/*v*) were added and incubated in a dark place at room temperature for 10 min. Plates were washed 3 times with 1% acetic acid and allowed to air dry overnight. Then, 150 μL of TRIS (10 mM) was added to dissolve the protein-bound SRB stain; the absorbance was measured at 540 nm using a BMG LABTECH^®^-FLUOstar Omega microplate reader (Ortenberg, Germany). Values for IC50 were determined using Origin 8.0 software (Origin Lab, Northampton, MA, USA). Dose–response curves were plotted after correction by subtracting the background absorbance from the controls. The relative cell viability (%) was calculated relative to the untreated control cells.

#### 2.6.3. In Vitro Scratch Assay

Cells were seeded at a density 3 × 10^5^ cells/well onto a coated 6-well plate for scratch wound assay and cultured overnight in 5% FBS-DMEM at 37 °C and 5% CO_2_ [46]. On the next day, horizontal scratches were introduced into the confluent monolayer; the plate was washed thoroughly with PBS, and control wells were replenished with fresh medium, while drug wells were treated with fresh media containing either NC-GL suspension or a 72 h extract of SC3-2 and SCGL3-2 in 5% FBS-DMEM. The plate was incubated at 37 °C and 5% CO_2_. Images were taken using an inverted microscope at indicated time intervals of 0, 24, 48, 72 and 96 h. Acquired images were analyzed by MII Image View software version 3.7.

### 2.7. In Vivo Wound Healing Studies

#### 2.7.1. Experimental Animals

In vivo studies were performed on Sprague Dawley rats according to the ethical guidelines of Alexandria University which comply with the National Institutes of Health guide for the care and use of laboratory animals (NIH Publications No. 8023, revised 1978). The study protocol was approved by the Institutional Animal Care and Ethics Committee of the Faculty of Pharmacy, Alexandria University, Egypt (approval code AU-06-2019/1/48, date of approval 30 April 2019). A total of 12 female rats weighing 200–250 g were separately housed at 22 ± 5 °C in a 12 h light/dark cycle. Rats were fed rodent chow and water ad libitum.

#### 2.7.2. Study Design

Rats were anesthetized using IP injection of ketamine hydrochloride (50 mg/kg body weight) and xylazine (5 mg/kg body weight) [38]. After removing the back hair of rats and disinfecting the shaved area, full-thickness round wounds with a diameter of 12 mm were developed by a sterile scalpel. For each animal, four wounds were created and treated as follows: untreated wound and treated with NC-GL dispersion, SC3-2 and SCGL3-2. No other topical medication was applied. Following treatment application, the wounds were covered with sterile dressing which was removed on day 9. Three time points were selected for animal sacrifice with 3–4 animals sacrificed at each time point (14, 21 and 28 days).

#### 2.7.3. Percentage Wound Closure

The change in open wound area was monitored using a digital camera on days 0, 3, 7, 14, 21 and 28, and the areas of wounds in each group were analyzed using Image J 2.0 software. Wound closure was expressed as percentage closure relative to the original wound and was calculated using the following formula:

$$Wound\;closure\;\left(\%\right)=\frac{A_{o}-A_{t}}{A_{0}}\times 100$$

where A_o_ is the initial area of the created wound, and A_t_ is the wound area at time t [47].

#### 2.7.4. Histopathological and Morphometric Assessment

Fourteen, twenty-one and twenty-eight days after wound induction, rats were euthanized by an overdose of ether, and the wound area was removed. Tissue samples were fixed in 10% formol saline for 24 h, dehydrated through a graded series of ethanol solutions, immersed in xylene, embedded in paraffin and sectioned in 5 μm-thick slices [48]. The deparaffinized sections were then mounted on glass slides, rehydrated and stained with hematoxylin and eosin (H&E) for detection of structural changes, extent of wound healing and formation of skin appendages, and with Masson’s trichrome stain for detection of collagen deposition and orientation of the collagen fibers. The stained sections were examined by a light microscope (BX41, Olympus Tokyo, Japan, Mic. Mag. ×100) coupled with a digital camera.

#### 2.7.5. Immunohistochemical Assessment

For detection of CD68-positive macrophages, deparaffinized sections obtained on day 7 [6] were placed on positively charged slides and rehydrated in descending grades of alcohol. The endogenous peroxidase activity was blocked using 3% hydrogen peroxide for 10 min. Following antigen retrieval, anti-CD68 primary antibody (mouse monoclonal Ab, clone KiM6, 1:100 dilution) and horseradish peroxide-conjugated secondary antibody (rat IgG, 1:100 dilution) were used. Microscopic evaluation and digital image analysis of CD68-positive immuno-reactivity using Image J software were conducted. Microscopic evaluation of the brown color of CD68-positive immuno-reactivity was conducted by a light microscope (BX41, Olympus Tokyo, Japan, Mic. Mag. ×200) coupled with a digital camera.

### 2.8. Statistical Analysis

Experiments were conducted at least in triplicate; data were expressed as mean ± standard deviation. Statistical analysis was carried out using SPSS^®^ software (SPSS, version 20; IBM, Chicago, IL, USA). One-way ANOVA test was used to compare between the different groups, followed by pairwise comparisons (Tukey test). The results were presented as the mean ± SD. A value of *p* < 0.05 was considered as statistically significant.

## 3. Results and Discussion

### 3.1. Formulation and In Vitro Characterization of Nanocrystals

Glibenclamide nanocrystals (NC-GL) were prepared using the bottom-up nanoprecipitation technique [33]. This process was tailored by varying several parameters to select the optimum formulation and processing parameters influencing the PS and PDI. These parameters included the effect of the stabilizer type and concentration, the drug/stabilizer ratio and the sonication time. DMSO was selected as the organic solvent of choice, showing the highest drug solubility (25 mg/mL) [34]. During the precipitation phase, a stabilizer was added to decrease the free energy, preventing aggregation and Ostwald ripening of the generated drug particles [49]. Stabilization has been explained by steric and/or electrostatic interactions, where stabilizer adsorption to the surface of the particles resulted in mutual repulsive forces and/or steric hindrance between the particles, respectively [34]. Based on this, various stabilizers were tested for NC-GL stabilization under similar experimental conditions including steric polymeric stabilizers (PVP K25, PEG 4000 and PVA), nonionic SAA (poloxamer188 and Kolliphor HS15) and an anionic SAA (SLS). The formulation design, PS and PDI are listed in Table 1.

PVP- and PVA-stabilized formulations showed a large PS exceeding 800 nm, with the PDI (0.48 and 0.63, respectively) indicating the polydispersity of the prepared nanocrystals. SLS-stabilized NC-GL, although possessing a similarly large PS, showed a significantly (*p* < 0.05) smaller PDI, which could be attributed to its anionic structure, resulting in high repulsive force between particles, hence preventing aggregation. A significant reduction in PS (*p* < 0.05) was obtained with poloxamer 188 and PEG 4000. The latter stabilized formulation showed higher monodispersity.

K-HS15, a third-generation nonionic SAA, was selected as the stabilizer of choice, showing the highest reduction in the PS and PDI (305 ± 12 nm and 0.24 ± 0.11, respectively). The effect of the probe sonication time on the PS and PDI was tested. Reducing the sonication time from 15 to 5 min resulted in a slight increase in PS, with no effect on the PDI (352.2 ± 2.2 nm and 0.29 ± 0.01).

Further investigations showed a slight reduction in PS with an increasing K-HS15 concentration from 0.1 to 0.2 % *w*/*v* (352 ± 2 and 339 ± 6 nm, respectively) while keeping the sonication time at 5 min. Likewise, the change in PS with an increasing drug-to-stabilizer ratio was not highly pronounced; thus, the lower SAA concentration (0.1% at a ratio of 1:20) was selected.

**Figure 1:**
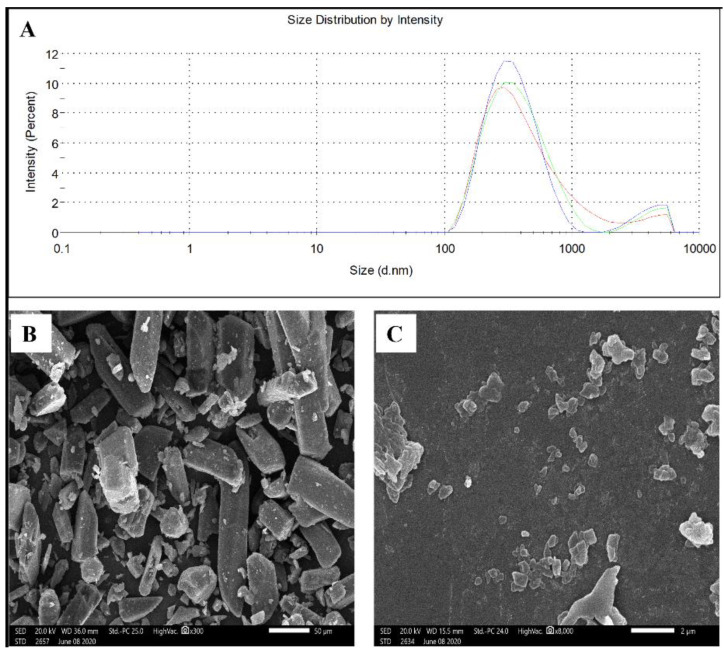
Size distribution by intensity curve of selected glibenclamide nanocrystal dispersion (NC-GL7) (**A**), and SEM images of glibenclamide coarse powder (**B**) and NC-GL7 (**C**) at magnifications ×300 and ×8000, respectively.

Based on the above results, NC-GL7 (352 ± 2, Figure 1A) was selected for further studies. A negative zeta potential (−22.4 mV) was observed for NC-GL7, ensuring good dispersion and preventing nanoparticle aggregation.

The GL coarse powder and the selected NC-GL formulation morphologies using SEM are shown in Figure 1B,C, respectively. GL was of an irregular shape with a broad size distribution (Figure 1B), whereas NC-GL showed plate-shaped crystals with a narrow size distribution comparable to that obtained by DLS (Figure 1C).

### 3.2. Assessment of Nanocrystals’ Solid State Properties

#### 3.2.1. Fourier Transmission Infrared Spectroscopy (FTIR)

**Figure 2:**
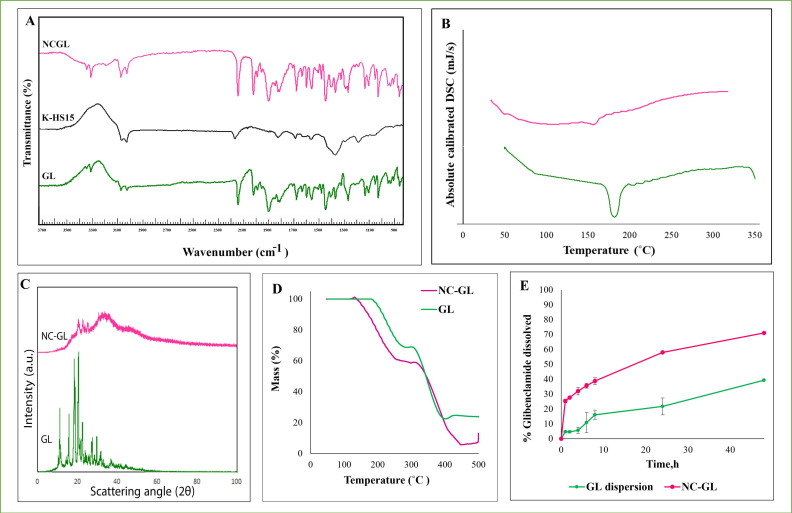
Crystallinity assessment of glibenclamide coarse powder and NC-GL: (**A**) FTIR spectra, (**B**) DSC thermograms, (**C**) PXRD profiles, (**D**) TGA thermograms and (**E**) in vitro glibenclamide coarse powder and NC-GL dissolution rate in phosphate buffer pH 7.4/CTAB 0.1% *w*/*v* at 37 °C over 48 h (*n* = 3).

As shown in Figure 2A, the GL coarse powder showed two characteristic peaks at 3365 and 3309 cm^−1^ corresponding to the amide stretching bands, a CH=CH band at 2929 cm^−1^, C=O stretching vibration at 1713 cm^−1^ and 1615 cm^−1^, C=C stretching bands at 1591 and 1519 cm^−1^ and symmetric and asymmetric S=O_2_ stretching at 1340 and 1155 cm^−1^, respectively [26]. Additionally, C–N, C–O and C–C had characteristic peaks at 1334, 1154, 924 and 810 cm^−1^. The K-HS15 spectrum presented two characteristic peaks at 2855 and 1094.2 cm^−1^ for C–H and C–O, respectively [50]. Other characteristic peaks were 1733 cm^−1^ for C=O stretching, and 724 cm^−1^ for C–H of alkanes [51]. Characteristic peaks for GL and K-HS15 were preserved in the NC-GL spectrum, reflecting the successful formulation and drug incorporation.

#### 3.2.2. Differential Scanning Calorimetry (DSC)

DSC thermograms of the GL coarse powder and NC-GL are shown in Figure 2B. The DSC thermogram of the GL coarse powder exhibited a single sharp endothermic melting peak at 181 °C [30]. The NC-GL DSC thermogram showed a minor shift in the melting peak to 167 °C. This could be a consequence of particle size reduction, which results in a greater fraction of more loosely bound molecules at the surface with less constrained thermal motion compared to larger particles [30].

#### 3.2.3. Powder X-ray Diffraction (PXRD)

PXRD was used to confirm the state of the drug crystallinity of the GL coarse powder and following nano-crystallization. As shown in Figure 2C, the GL coarse powder displayed characteristic sharp and intense diffraction peaks at 2θ values of 11.33°, 15.85°, 18.53°, 20.69°, 22.77°, 27.57°, 30.08° and 31.9°, indicating its crystalline nature [26,30]. The precipitation and nano-crystallization of GL to form NC-GL did not interfere with the GL crystalline state as the diffraction patterns for GL were preserved at 2θ values of 20.84°, 22.92° and 30.08° but with a lower intensity, reduced sharpness and slight broadening of peaks (Figure 2C). These spectral changes were previously reported and attributed to the “particle size broadening” phenomenon associated with PXRD profiles of small-sized crystalline materials [30,52].

#### 3.2.4. Thermal Gravimetric Analysis

The TGA thermograms of the GL coarse powder and NC-GL (Figure 2D) showed that GL is thermally stable up to 185 °C. The TGA curves indicated mass loss in well-defined stages between 185 and 500 °C. A mass loss of 31.2% was observed for the first stage (185–280.4 °C). The second stage of decomposition (280.4–390.3 °C) involved a loss in mass of 44.7%. Attia et al. [53] suggested the mass loss to correspond to the elimination of C_7_H_13_N_2_O and C_10_H_11_NClO_2_ for stages 1 and 2, respectively. NC-GL also showed mass loss in stages, but with the first stage mass loss starting at a lower temperature (140–282 °C), and a higher percentage of loss for stages 1 and 2 (40 and 52.2%, respectively). Such difference in thermal stability could be attributed to the smaller particle size and increase in the interface of NC–GL, allowing for a faster heat absorption and dissipation [54].

### 3.3. In Vitro Dissolution of Nanocrystals

The effect of nano-crystallization on the dissolution rate of GL was studied using the dialysis method (Figure 2E). Based on the results of a solubility study, phosphate buffer pH 7.4 containing 0.1% *w*/*v* CTAB, showing the highest GL solubility (450 µg/mL), was selected as the dissolution medium to achieve the sink condition.

A significant enhancement in drug dissolution (*p* < 0.05) was observed for the NC-GL compared to the GL powder dispersion at all time intervals studied (Figure 2E). A 57 ± 0.6% dissolution compared to 21.76 ± 5.67% was achieved by NC-GL compared to the GL coarse powder after 24 h (2.6-fold increase in drug dissolution). An enhancement in drug dissolution was previously reported by Hany et al. [30], where within 24 h, about 85% of the GL content was released from nano-GL compared to 61% from micro-GL. This could be attributed to the effect of the stabilizer on the NC formulation, enhancing both drug dissolution and formulation stabilization [55]. Moreover, it was proclaimed that the dissolution rate of drug nanocrystals is proportional to the particle size reduction and hence the available surface area for dissolution [33].

### 3.4. Preparation of Collagen/Chitosan Scaffold

CO/CS scaffolds were prepared using the freeze drying technique, which offers the advantage of employing water upon freezing as a porogen [56]. Freeze drying removes the frozen water/solvent from the polymer by sublimation under vacuum, leading to an almost anhydrous 3D polymer structure [57]. This is beneficial in creating a porous sponge such as a scaffold with a large surface/volume ratio, providing sufficient space for cell growth and proliferation, thus accelerating the healing process [58].

The collagen/chitosan ratio and total polymer content were varied to obtain a scaffold with the most favorable properties regarding porosity and swelling behavior. Formulations are listed in Table 2.

It is well reported that crosslinking confers strength and toughness to biomaterials [59]. Crosslinking has been designated as an important strategy in scaffold fabrication, yielding porous structures with water retention ability [59]. In the current work, glutaraldehyde (GA) was used as a chemical crosslinking agent at a concentration of 0.1% *w*/*w* of the total polymer weight [1]. GA’s safety at the used concentration of 0.1% *w*/*w* on skin fibroblasts is well established [1,60]. The GA structure comprises –OH and –COOH groups. These are capable of reacting with the –NH_2_ or –OH groups of collagen, improving stability and increasing resistance to degradation against collagenase activity [61]. Due to its large number of amino groups, chitosan can function as a bridge to increase the crosslinking efficiency of GA in CO/CS scaffolds [1]. Moreover, activation of its glucuronic acid residues’ carboxyl groups allows for the formation of amide bonds with collagen [1,59].

### 3.5. Scaffold Porosity and Pore Size

Scaffold porosity was evaluated by the alcohol displacement method (Table 2). For blank scaffolds, no significant change (*p* > 0.05) in porosity was observed with an increasing total polymer content from 3 to 6% while maintaining the same collagen/chitosan ratio. Increasing the collagen content to 50 and 67% resulted in a significant increase in porosity (*p* < 0.05) compared to the scaffolds containing 33 and 20%. This is in accordance with previous investigations demonstrating the influence of collagen on the rearrangement of chitosan chains, providing 3D structures with larger pores [60].

High scaffold porosity offers the benefit of exudate absorption from the wound surface [62]. It also allows for the transfer and exchange of nutrients and oxygen to the cells, which is essential for cell proliferation and vascularization, consequently facilitating the healing process [31]. The porous scaffolds could serve as a matrix for the ingrowth of cells into the wound and boost tissue regeneration [39,62]. In this context, blank scaffolds showing the highest porosity (SC3-1, SC3-2, SC6-1 and SC6-2) were selected for NC-GL loading.

NC-GL-loaded scaffolds showed a significant increase in porosity (*p* < 0.05) compared to their blank counterparts for the total polymer content of 3% (69 ± 5 and 68 ± 6 for SC3-1 and SC3-2 compared to 91 ± 4 and 94 ± 4 for SCGL3-1 and SCGL3-2, respectively). This increase could be attributed to the physical interaction between the polymer chains and embedded nanoparticles in the core resulting in the formation of anchor point-like structures that restrict polymer chain movements upon freeze drying, resulting in larger pores [63]. This postulation was supported by the pore size measurements obtained by SEM and showing an increase in pore size upon NC-GL incorporation from 133 ± 19 to 288 ± 39 µm for SC3-1 and from 141 ± 40 to 221 ± 61 for SC3-2. This highly porous nature is expected to improve drug release at the wound site while offering mechanical interlocking between scaffolds and the surrounding tissue, thus improving the scaffold physical stability.

On the other hand, no significant change in porosity (*p* > 0.05) was observed following nanocrystal embodiment for scaffolds with a high total polymer content (SC6-1 and SC6-2). Despite possessing similar porosities, a highly significant reduction in pore size was observed for SCGL-2 compared to SC6-2 (204 ± 34 and 93 ± 13 µm for SC6-2 and SCGL6-2, respectively). For SC6-1, no significant change (*p* > 0.05) in pore size was observed upon NC-GL loading.

### 3.6. Scanning Electron Microscopy (SEM)

**Figure 3:**
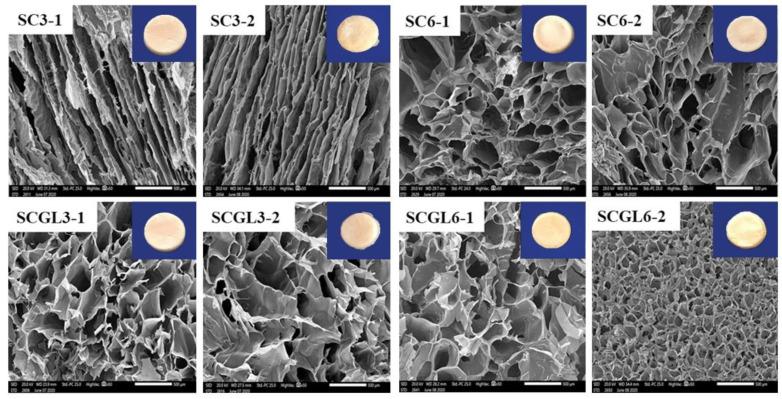
SEM micrographs of cross-sections of selected scaffolds (at magnification 50×).

The influence of the composition on the scaffold structure and pore shape was studied by SEM. The cross-section morphologies of the selected CO/CS scaffolds (SC3-1, SC3-2, SC6-1, SC6-2, SCGL3-1, SCGL3-2, SCGL6-1, SCGL6-2) are shown in Figure 3. All scaffolds revealed a highly interconnected structure which is classical for scaffolds obtained by freeze drying [58] due to the formation of ice crystals binding the polymer in inter-crystalline spaces to form pore walls [58]. Moreover, the CO/CS mixture resulted in a homogeneous porous structure with a narrow size distribution of the pores for most of the prepared scaffolds, similar to previous reports [58]. Further, non-excessive crosslinking could be efficiently indicated from the pore interconnectivity [64]. Scaffolds also showed a smooth and homogeneous pore wall surface.

It was observed that the pore shape tended to be rounded in all scaffolds, yielding a sponge-like structure, except for SC3-1 and SC3-2, where the pores were channel-shaped. This could be the result of a slight collapse of the scaffold during freeze drying [65]. NC-GL loading into these scaffolds prevented collapse and allowed for the formation of rounded interconnected pores which is favorable for vascularization and cellular colonization. While retaining the sponge-like structure, SCGL6-2 showed significantly small pores compared to SC6-2. The pores were shown to be symmetrical, as indicated by the insignificant difference in the pore size of the longitudinal and transverse sections (*p* > 0.05).

### 3.7. Solid State Properties of CO/CS Scaffolds

#### 3.7.1. Fourier Transmission Infrared Spectroscopy (FTIR)

The FTIR spectrum of collagen displayed characteristic intense absorption bands at 1626 cm^−1^ originating from the C=O stretching vibration of amide I, 1523 cm^−1^ for the N–H bend of amide II, 1238 cm^−1^ for amide III and other bands at 3290 cm^−1^ for O–H stretch [16]. On the other hand, chitosan showed peaks at 3413 cm^−1^ for the O–H stretching band, 2866 cm^−1^ for C–H stretching and around 1599 cm^−1^ for the amide I band. Moreover, between 1058 and 1022 cm^−1^, a C–O stretching signal was observed. Peaks at 2923 and 2880 cm^−1^ are associated with the methylene groups, and signals in the 1200–1000 cm^−1^ range are characteristic of the saccharide structure [66,67].

**Figure 4:**
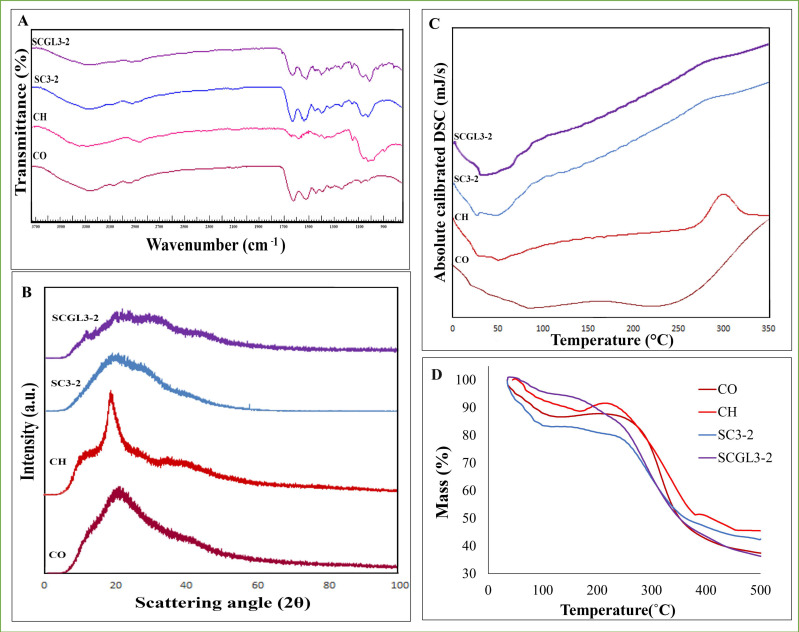
Solid state properties of CO, CS and their composite scaffolds: (**A**) FTIR spectra, (**B**) PXRD profiles, (**C**) DSC thermograms and (**D**) TGA thermograms.

The spectrum for the SC3-2 scaffold was obtained. The spectrum showed the characteristic peaks of the pure components, with intensities approximately proportional to the mass fraction of each component, as it can be observed in Figure 4A. The spectrum is not a simple superimposition of the spectra of the separated components due to the interactions between collagen and chitosan by the formation of inter- and intra-macromolecular hydrogen bonds before GA addition and also the formation of covalent bonds resulting from crosslinking [58]. These observations were previously attributed to the electrostatic interactions between the positively charged NH_3_^+^ in CS and the negatively charged COO^−^ in collagen [16]. The interaction is confirmed by the slight shifting of the amide I absorption band at 1599 cm^−1^ displayed by the FTIR spectrum of the CS scaffolds in the direction of the band exhibited by FTIR of CO at 1523 cm^−1^, showing a peak at 1538 cm^−1^ [60]. The spectrum for SCGL3-2 showed similar peaks to SC3-2, with GL characteristic bands preserved but with lower intensities, proving the successful encapsulation of NC-GL in the scaffolds.

#### 3.7.2. Powder X-ray Diffraction (PXRD)

The PXRD patterns of collagen, chitosan and the selected scaffolds (SC3-2 and SCGL3-2) are illustrated in Figure 4B. PXRD of pure chitosan displayed a strong diffraction peak at 2θ = 19.79° [68], while collagen showed one broad peak at 2θ = 22°. This broad peak in collagen is related to the diameter of the triple-helix chain of the collagen molecule [69]. The absence of sharp peaks in the tested materials is attributed to their amorphous nature [69]. In SC3-2 and SCGL3-2, the diffraction peaks of collagen and chitosan were preserved but with a lower intensity. Moreover, the PXRD curve of SCGL3-2 showed low-intensity peaks at 11.33° and 27.57°, which are specific for GL.

#### 3.7.3. Differential Scanning Calorimetry (DSC)

As shown in Figure 4C, the DSC thermograms of collagen and chitosan revealed characteristic endothermic peaks at 108 °C and 90 °C, respectively, associated with the loss of bound water with the hydrophilic moieties. Another substantial peak of collagen was observed at 263 °C due to denaturation and conversion of the triple helix of proteins to fibrils as a result of the thermal degradation of the collagen molecules [69,70]. An exothermic peak at 309°C was shown for chitosan corresponding to its degradation by depolymerization [32].

DSC analysis of SC3-2 showed a primary peak at 85.2 °C and a secondary peak at 287.5 °C, confirming the merging of the peaks of biopolymeric components [9]. The thermogram of the selected scaffold proves the formation of a new crosslinking complex between the ingredients [69,71]. The peaks were preserved but slightly shifted in the SCGL3-2 thermogram, where the primary peak appeared at 71.2 °C. This could be attributable to the effect of NC-GL on the scaffold structure and hence the thermal behavior, as observed by the significantly increased porosity and pore size compared to SC3-2, which could reflect loosening of the polymeric matrix, allowing for easier loss of water of hydration. The disappearance of the NC-GL peak could result from the low drug amount not being high enough to produce sensitivity [72].

#### 3.7.4. Thermal Gravimetric Analysis

The TGA thermograms of SC3-2 and SCGL3-2 shown in Figure 4D proved their thermal stability across the high temperature range. The first low percentage weight loss was due to loss of structurally bound water (17.5 and 12.5% in the ranges 36.9–158.4 and 44–134 °C for SC3-2 and SCGL3-2, respectively). Above this temperature range, mass loss was corelated with the degradation of collagen and chitosan (41.2 and 58.9% for SC3-2 and SCGL3-2, respectively). SCGL3-2 showed a higher percentage of mass loss, which could be due to the presence of NC-GL.

### 3.8. Swelling Behavior and Water Holding Capacity

**Figure 5:**
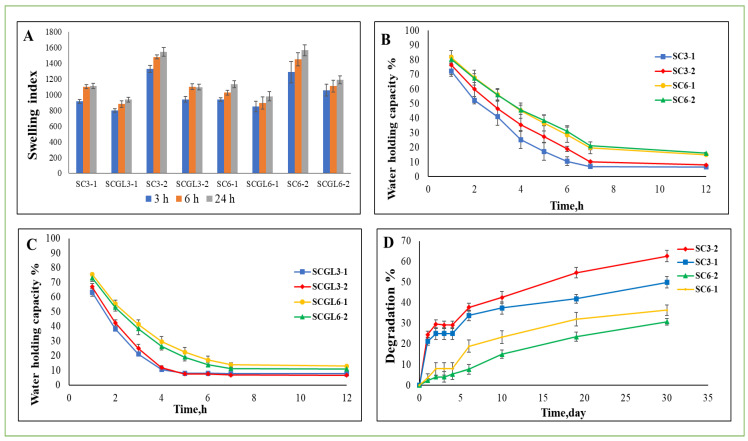
Selected scaffolds’ swelling index at different time intervals (3, 6 and 24 h) (**A**), water holding capacity for blank (**B**) and NC-GL-loaded scaffolds (**C**) and degradation percentage over 30 days (**D**), *n* = 3.

The swelling properties of the scaffolds with various polymer ratios before and after incorporation of NC-GL, immersed in PBS (pH 7.4), were investigated at 3, 6 and 24 h (Figure 5A). A slight change in swelling index (SI) values was observed over the studied time intervals. Since no significant (*p* > 0.05) increase in the SI was observed between the 6 and 24 h time intervals, SI values at 24 h were considered as the maximum swelling ability and are listed in Table 2. All tested scaffolds showed a high SI ranging from 507 ± 136 to 1565.59 ± 68.29% which is important for decreasing wound maceration and accelerating healing. Moreover, eliminating excess exudates keeps the wound dry, thereby preventing infection [73]. The water-binding ability of the CO/CS scaffold could be attributed to its hydrophilicity and the ability to maintain its 3D structure [65]. Increasing the total polymer content from 3 to 6% *w*/*v* while maintaining the ratio between collagen and chitosan did not significantly affect the SI. On the other hand, a significant increase in the SI (*p* < 0.05) was observed with an increasing collagen/chitosan ratio, where for the selected scaffolds, a 1.4-fold increase in the SI was achieved with increasing collagen from 50 to 67%. This could be explained by the abundance of collagen hydrophilic groups that are not utilized for chitosan crosslinking [74]. A similar pattern was obtained for scaffolds loaded with NC-GL, but with a significantly lower SI (*p* < 0.05) for the loaded scaffold compared to its blank counterpart. Despite the increase in porosity achieved following NC-GL loading in scaffolds with a total polymer content of 3% *w*/*v*, NC-GL might have provided a longer water diffusion path, hindering the loosening of polymeric chains and resulting in a lower swelling percent [75]. In addition, the lower SI of the loaded scaffolds could be attributed to the lower hydrophilicity of NC-GL relative to the polymers used [76] and the possibility of mechanical interlocking between polymeric chains created by incorporation of NC-GL [64,77], where NC might have acted as an additional physical crosslinker preventing water absorption [75].

Maintaining a moist environment around a wound can accelerate recovery and decrease the risk of systemic infection [78]. Moreover, a scaffold with a high water holding capacity helps in absorbing wound exudates, thus facilitating healing. At the end of the swelling experiment, the water holding capacity of the swollen scaffolds was evaluated (Figure 5B,C) by calculating the percentage weight remaining over time. Scaffolds with a higher polymer content (6% *w*/*v*), whether blank or loaded with NC-GL, possessed a water holding capacity exceeding that for scaffolds with a total polymer content of 3% *w*/*v* over time. On the other hand, no significant difference (*p* > 0.05) was observed upon varying the collagen/chitosan ratio. Moreover, NC-GL incorporation in the scaffolds decreased their water holding capacity, where for scaffolds with a 3% polymer content, water was lost and a plateau was reached, with no further loss until the end of the study after 7 and 5 h for the blank and loaded scaffolds, respectively, whereas for scaffolds with a 6% polymer content, water was lost after 7 h for the NC-GL-loaded scaffolds, while the blank scaffolds still retained 21% of their water content. This is in accordance with their swelling behavior, where drug loading decreased the potential for water absorption.

### 3.9. In Vitro Biodegradation

Biodegradation is a time-dependent process and is one of the critical properties of ideal biodegradable scaffolds for wound healing. Non-biodegradable biomaterials may cause a long-term inflammatory reaction after in vivo implantation, leading to delayed healing [12]. Enzymatic degradation of chitosan/collagen scaffolds was studied by monitoring the residual mass percent of the samples after several days of incubation with lysozyme in phosphate buffer solution (pH = 7.4) at 37 °C to mimic physiological conditions. Chronic wounds, especially diabetic wounds, are characterized by the presence of proteases including lysozyme [79] which is an enzyme that specifically degrades β-1,4-glycosidic linkages between N-acetyl glucosamine and glucosamine in chitosan [79].

The degradation profiles of the blank scaffolds with different polymer ratios are shown in Figure 5D. Preliminary studies on the loaded scaffolds showed a non-significant effect of NC-GL loading on in vitro degradation, and therefore the loaded scaffolds were not included in the study for simplification. The results prove the success of the method used to fabricate scaffolds in a way that they can resist rapid degradation upon contact with a moisturized surface. The use of GA for crosslinking and the chitosan content of the scaffold which might also function as a crosslinking bridge [61] are indispensable for improving scaffold biostability.

High-polymer content scaffolds (SC6-1 and SC6-2) were significantly (*p* < 0.05) more resistant to digestion, showing the lowest values of mass loss following incubation. For scaffolds with the same total polymer content but with varying collagen/chitosan ratios, there was no significant difference in the degradation value (*p* > 0.05) for the first 4 days of the study. Starting from day 6, percentage weight loss differed significantly (*p* < 0.05), but with variation in the pattern for both the 3 and 6% total polymer contents. For the 3% total polymer content, SC3-2 degradation outvalued that of SC3-1. This could be attributed to the lower chitosan content being acted upon by lysozyme in addition to the higher proportion of the hydrophilic polymer; collagen facilitated water absorption, as reflected by the higher SI, thus allowing lysozyme penetration into the scaffold. On the contrary, SC6-1 showed higher mass loss compared to SC6-2. This could be explained by the significantly larger pore size allowing lysozyme to freely interact with the polymer chains, thus increasing scaffold degradation [80].

### 3.10. Mechanical Properties

**Figure 6:**
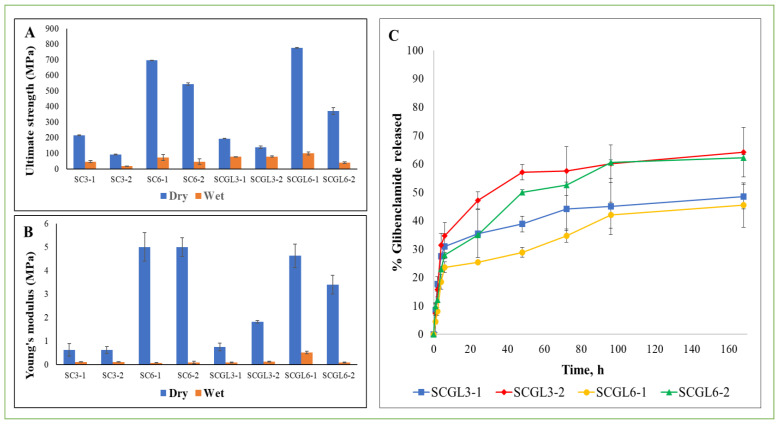
Mechanical properties of bioactive CO/CS scaffolds in wet and dry states expressed by (**A**) ultimate strength and (**B**) Young’s modulus. (**C**) In vitro percentage glibenclamide release from different CO/CS scaffolds in phosphate buffer pH 7.4/CTAB 0.1% *w*/*v* at 37 °C over 7 days.

Suitable mechanical properties could prevent scaffold destruction via mechanical transduction. Moreover, for the healing cascade, mechanical properties are important for supporting cell penetration, migration and proliferation [12]. The ultimate compressive strength and Young’s modulus of the selected scaffolds in both dry and wet states were tested and are presented in Figure 6A,B.

The mechanical properties of the scaffolds were affected by the total polymer content, collagen/chitosan ratio and incorporation of NC-GL, in addition to scaffold wetting. Figure 6A shows that for the dry blank scaffolds, increasing the total polymer content from 3 to 6% *w*/*v* while maintaining the ratio of collagen to chitosan resulted in a highly significant (*p* < 0.05) increase in the ultimate compressive strength (215.85 ± 12.9 and 696 ± 80.6 MPa for SC3-1 and SC6-1, respectively, and 92.16 ± 2.3 and 544 ± 9 MPa for SC3-2 and SC6-2, respectively) and Young’s moduli (0.63 ± 0.26 and 5 ± 0.61 N/mm^2^ for SC3-1 and SC6-1, respectively, and 0.62 ± 0.14 and 4.99 ± 1.33 N/mm^2^ for SC3-2 and SC6-2, respectively). The results also show that increasing the chitosan ratio relative to collagen was also accompanied by an increase in the ultimate compressive strength, which was more pronounced in the blank scaffolds with a lower total polymer content (2.34- and 1.27-fold increase for polymer contents of 3 and 6% *w*/*v*, respectively). Previous studies suggested chitosan to act as a binder for collagen fibrils, enhancing interfibrillar bonding and leading to a more efficient collagen fibril network [12,58] through formation of internal, hydrogen-bonded polymeric networks between collagen and chitosan [40], thus promoting the mechanical stabilization of the matrix.

NC-GL loading into scaffolds resulted in an insignificant change (*p* > 0.05) in the mechanical strength for the scaffolds with a collagen/chitosan ratio of 1:1 (SC3-1 and SC6-1). On the other hand, the SC3-2 scaffold showed a significant (*p* < 0.05) increase in the ultimate compressive strength (92.19 ± 2.3 and 138.67 ± 8.6 MPa for SC3-2 and SCGL3-2, respectively) and Young’s moduli (0.62 ± 0.144 and 1.82 ± 0.05 N/mm^2^ for SC3-2 and SCGL3-2, respectively) upon loading of NC-GL. This could be attributed to the NC-GL reinforcement effect exerted on the CO/CS scaffolds. This reinforcement effect was explained to be due to the mechanical interlocking formed between the polymer chains and the integrated nanoparticles upon freeze drying [81]. This anchor point-like structure developed imparts with higher resistance to deformation [64]. This effect was not obvious in the scaffolds with a higher chitosan content, possibly due to the highly pronounced effect of chitosan on the scaffold mechanical strength masking the effect of NC-GL. The SCGL6-2 scaffold’s mechanical strength, although relatively high (371 ± 21.2 MPa), was significantly lower than that of SC6-2. This could be attributed to the significantly smaller pore size of SCGL6-2 compared to SC6-2 (Table 2). Likewise, hyaluronan-collagen scaffolds showed a decrease in mechanical strength with a decrease in pore size, which was explained by the change from cubical to spherical shapes of the pores as well as the more homogeneous structure when increasing the pore size [82]. Although the mechanical strength of the scaffolds was reduced by wetting, which partially simulates exudate absorption, the value of Young’s modulus (83–519 KPa) was still in the range accepted for scaffolds applied for wound healing as it was previously reported that Young’s modulus of the skin varies from 10 kPa to 50 MPa, and hence scaffolds’ mechanical strength should lie in this range [83].

For all scaffolds examined, a significant decrease (*p* < 0.05) in ultimate compressive strength and Young’s moduli was observed following scaffold wetting in saline. This is attributable to the presence of water acting as a natural plasticizer for both collagen and chitosan [58].

The mechanical strength values obtained show that the prepared scaffolds could resist deformation and possess sufficient strength when applied as a wound dressing.

### 3.11. In Vitro Drug Release

The in vitro drug release of GL from SCGL3-1, SCGL3-2, SCGL6-1 and SCGL6-2 was investigated in phosphate buffer pH 7.4/CTAB 0.1% *w*/*v* at 37 °C over 7 days. The release profiles are shown in Figure 6C.

For all scaffolds, a biphasic release profile was observed with a moderate burst during the first 6 h (23.63–34.64%) followed by a sustained release over the study period (45.53–64.13% on day 7). This could be explained by the initial swelling followed by the slower degradation of the CO/CS matrix [14]. The hydrophilic nature of collagen and chitosan allowed the permeation of water into the polymer matrix and diffusion of GL from NC-GL. Previous studies showed that bulk-eroding polymers are often characterized by a burst drug release during the first few hours of incubation, followed by a slow, diffusion-controlled release [84]. Moreover, scaffold preparation by freeze drying yielded highly porous scaffolds, promoting exposure of a large surface to the release medium [63].

The total polymer content proved to have a significant effect on the drug release profile, where a significant reduction in percentage cumulative drug released (*p* < 0.05) was observed for scaffolds with a total polymer content of 6% compared to 3% *w*/*v* over the first 48 h of the study period. The slow drug release from scaffolds containing a higher polymer content could be attributed to the formation of a more viscous diffusion layer that delays drug release from the polymer matrix [69].

Furthermore, increasing the collagen/chitosan ratio resulted in a significant increase in GL release at all time points (*p* < 0.05). This could be explained by the high collagen hydrophilicity, resulting in an increased swelling potential, consequently facilitating drug release. Additionally, in the scaffolds with a 1:1 collagen/chitosan ratio (SCGL3-1 and SCGL6-1), the strong synergistic interaction between polymers might have led to the formation of a tighter network retarding drug diffusion [85]. The sustained GL release maintained by the CO/CS scaffold is beneficial for effective prolonged drug release at the wound site and for preventing rapid drug clearance from the wound bed [86].

Based on the results of the above experiments, the scaffolds with a lower total polymer content (3%) and higher collagen/chitosan ratio (2:1) were selected for further evaluation of in vitro hemocompatibility and biocompatibility and in vivo efficacy. SCGL3-2 showed a high SI with an acceptable water holding capacity. These were reported to be of benefit in absorbing exudates while keeping the wound moist. Additionally, its high porosity and large pore size are expected to allow for cell proliferation and exchange of waste and nutrients. Moreover, the high mass loss observed and fast drug release rate relative to the other scaffolds could be beneficial, allowing a rapid availability of the drug at the wound site.

Release kinetics were determined by plotting the release data over the study period according to first-order, Higuchi diffusion, Korsmeyer–Peppas, Hixson–Crowell and Baker–Lonsdale equations. The greatest correlation coefficient (r) and lowest mean standard error (MSE) (Supplementary Materials Table S1) were used as statistical parameters to designate the function that the data best fit. The results are indicative of diffusion-controlled GL release as the greatest r value (mean value 0.95) and the minimum MSE value were observed for the Korsmeyer–Peppas model. The calculated n-values for all examined formulations were less than 0.5, indicative of Fickian diffusion.

### 3.12. Hemocompatibility Evaluation

The hemocompatibility of the selected scaffolds with a total polymer content of 3% and a CO/CS ratio of 2:1 was tested. Scaffolds with a 6% total polymer content were included in the study for comparison.

#### 3.12.1. Protein Adsorption

Protein adsorption is an indicator of the blood compatibility of biomaterials and a determinant of the mechanism and degree of intrinsic thrombosis [87]. Plasma protein interactions with surfaces trigger a blood coagulation cascade [42]; thus, an enhancement in protein adsorption signifies a better thrombotic property [75]. BSA adsorption to the surface of the blank and NC-GL-loaded scaffolds was studied on selected scaffolds (Table 3). Increasing the total polymer content did not significantly (*p* > 0.05) affect the degree of protein adsorption (9.58 ± 1.19 and 8.78 ± 0.11 mg/g for SC3-2 and SC6-2, respectively). Incorporation of NC-GL into the scaffold resulted in a 1.8-fold increase in protein adsorption. In general, surfaces with lower hydrophilicity show higher protein adsorption capabilities due to the easier displacement of bound water molecules [43]. Moreover, the surface topography might influence the amount of protein adsorbed [42]. As the dimensions of proteins are in the nanometer range, nanoscale topographies are thought to influence protein behavior [42]. Similar findings were observed for nZnO-loaded hydrogels showing increased BSA adsorption on hydrogel samples by increasing the amount of nZnO, attributed to the reduced hydrophilicity compared to the hydrogel matrices (PVA and CS) [43]. Likewise, addition of hydroxyapatite nano-crystallites modified the characteristics of the chitosan scaffold surfaces (e.g., chemical composition, electric charge and morphology), thereby greatly increasing the quantity of active sites on the composite scaffolds for possible interactions with proteins [88].

#### 3.12.2. Whole Blood Clotting

The hemostatic potential of the studied scaffolds is shown in Table 3. After contacting the samples with whole blood, RBCs that were not trapped in the clot were hemolyzed with distilled water. The absorbance value of the resulting hemoglobin solution was noted at 545 nm. Large OD values indicate a large amount of hemolyzed hemoglobin, inversely correlating with clot formation [89]. The results suggest that the low-polymer content scaffolds had significantly higher OD values (*p* < 0.05), having a high antithrombogenic effect (0.78 ± 0.09 and 0.35 ± 0.04 for SC3-2 and SC6-2, respectively). A similar pattern was observed for the drug-loaded scaffolds (0.63 ± 0.17 and 0.2 ± 0.02 for SCGL3-2 and SCGL6-2, respectively). It was noted that collagen can promote blood clotting due to its intact triple helix and excellent hygroscopic properties [90]. Collagen crosslinking supports a dense network microstructure and changes its surface charge, resulting in a higher blood clotting index [11]. Additionally, chitosan acts as a blood coagulant, promoting platelet adhesion and aggregation. Additionally, its positively charged groups interact with the negatively charged red blood cell membranes [91]. Incorporation of NC-GL resulted in a significant (*p* < 0.05) reduction in OD values for scaffolds with a 6% *w*/*v* total polymer content. This is in accordance with the higher degree of protein absorption. The surface charge of incorporated NC-GL could have effectively activated platelets and accelerated fibrin formation [92]. Moreover, SCGL6-2 showed a significantly smaller (*p* < 0.05) pore size compared to SC6-2. Scaffolds with a smaller pore size were shown to achieve faster clotting and stable clots. Clotting on chitosan scaffolds indicated that the smaller pore size showed fibrin fibers “bridging” the pores’ walls and resulting in faster clotting compared to the larger pores [93].

#### 3.12.3. In Vitro Hemolysis Assay

Hemolysis, defined as the release of hemoglobin into plasma due to damage of erythrocytes, was directly pertinent to the blood compatibility of the materials [2]. Preservation of erythrocytes’ normal physiological state when contacting with scaffolds is essential for vascular scaffold application. The hemolysis assay evaluated the destructive potential of the scaffolds through determining the hemoglobin from broken erythrocytes [94].

As it can be observed in Table 3, the in vitro hemolytic activity of all samples was lower than that of the positive control (100% hemolysis). Increasing the total polymer content resulted in a significant increase in percentage hemolysis. On the other hand, incorporating NC-GL into the scaffolds resulted in a significant reduction in hemolytic activity (*p* < 0.05), with SCGL3-2 being non-hemolytic (0.22%) as per the American Society for Testing and Materials classification [95]. The results of the hemocompatibility studies confirm the convenience of testing the potential of SCGL3-2 for wound healing.

### 3.13. In Vitro Biocompatibility

#### 3.13.1. Cell Viability Assay

The effect of GL on HSF cells’ viability was studied using the SRB assay following 72 h of incubation. Serial dilutions of GL and NC-GL in the concentration range of 0.02 to 202.4 µM were tested. The IC_50_ values were calculated from the dose response curves of GL and NC-GL. Nano-crystallization resulted in a slight increase in the IC50 of GL from 53.64 to 79.35 µM. The safety and biocompatibility of CO/CS scaffolds crosslinked with GA are well reported in the literature [1] and thus were not tested in the current study.

#### 3.13.2. In Vitro Scratch Assay

**Figure 7:**
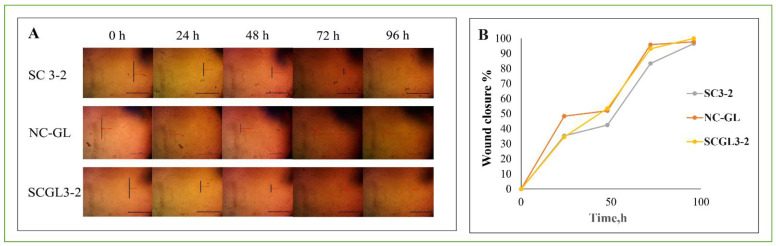
In vitro wound healing assay on HSF cells: (**A**) inverted microscope images showing measurements of cell migration (Scale bar = 1 mm), and (**B**) wound closure percentage at different time intervals (24, 48, 72 and 96 h) (*n* = 3).

The in vitro scratch assay is considered as an essential biomarker to estimate the migration and proliferation potential of cells across a wound following treatment [46]. Fibroblasts are stimulated to migrate into the wound by PDGF, fibroblast growth factor (FGF), TGF-β and epidermal growth factor (EGF) [96]. The effect of NC-GL dispersion and the selected scaffold SCGL3-2 on the extent of cell regrowth to close the scratch wound was measured after 0, 24, 48, 72 and 96 h of incubation (Figure 7). The results indicate good in vitro wound healing of the tested formulations, probably due to the well-reported effect of GL on increasing the expression of several pro-healing growth factors such as IGF-1 and TGF-β [24], which stimulates collagen secretion and inhibits different MMPs, promoting collagen fiber accumulation [97]. Pretreatment of mesenchymal stem cells with TGF-β1 in vitro resulted in a sustained improvement in cell migration and adhesion [98]. Collagen was previously shown to promote cell proliferation and cell survival under stress, in addition to promoting high cell adhesion to the cell culture surface [78,99,100]. After 72 h, the whole artificial wound area was fairly filled with proliferating cells (95.87 ± 7.2, 83.37 ± 12.7 and 93.07 ± 8.3% for NC-GL, SC3-2 and SCGL3-2, respectively). No statistically significant difference was observed between the tested formulations. The slight retardation in cell regrowth observed for SCGL3-2 compared to NC-GL at 24 h could be attributed to the chitosan content, which has been previously shown to decrease the proliferation of the tendon sheath fibroblasts [101].

### 3.14. In Vivo Wound Healing Studies

#### 3.14.1. Macroscopic Observations

**Figure 8:**
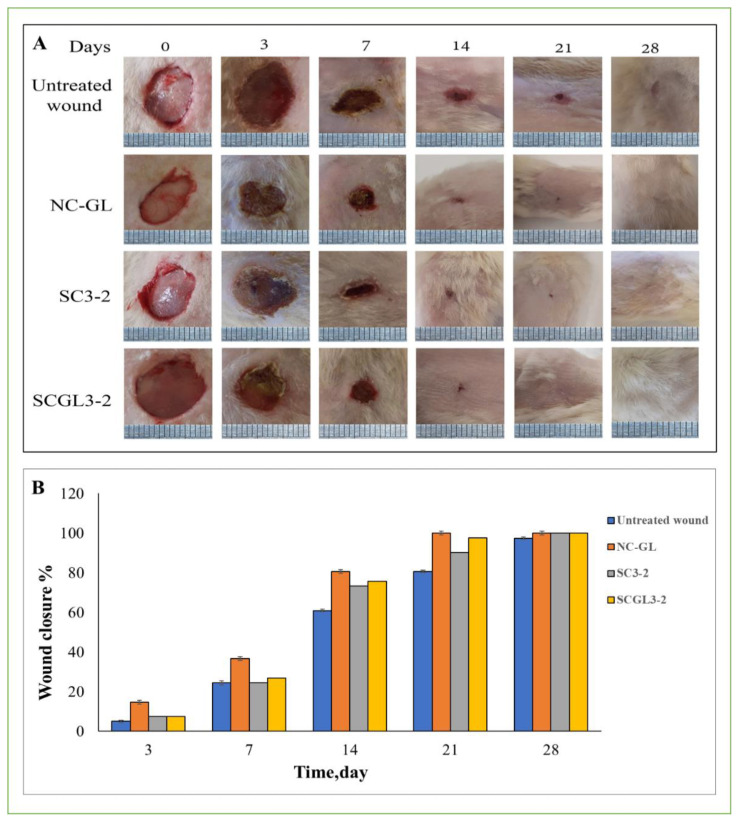
In vivo wound healing study: (**A**) digital images of wounds over 28 days, and (**B**) percent of wound closure on days 3, 7, 14, 21 and 28 (*n* = 3–12).

The wound healing efficiency of NC-GL either as a dispersion or loaded into the CO/CS scaffold (SCGL2-3) was evaluated via an in vivo full-thickness wound model and quantified with excision wound area image analysis. The aim of wound treatment is to accelerate the healing time and avail undesired complications such as scarring [102]. The photographs of the wounds at 0, 3, 7, 14, 21 and 28 days post-excision in terms of wound appearance and change in wound area following treatment are represented in Figure 8A. The recorded wound size and the percentage of wound closure were determined and compared among all experimental groups (Figure 8B).

Over the wound healing period, pronounced differences in percentage wound closure were observed between the treated and untreated control wounds. On days 3 and 7 post-excision, wounds treated by the NC-GL dispersion showed a significantly higher percentage wound closure compared to SC3-2 and SCGL3-2 (*p* < 0.05) owing to NC-GL dissolution, which is faster than the drug release from the scaffold. Starting from day 14, the rate of wound closure of SCGL3-2 increased, showing a non-significant difference compared to NC-GL (*p* > 0.05). Additionally, all wounds were re-epithelized except for the untreated wound. Complete wound closure was achieved by NC-GL and SCGL3-2 on day 21, whereas for SC3-2 and the untreated control groups, wound closure was only achieved on day 28. Essentially, the regenerated skin at full-thickness wounds treated with NC-GL and SCGL3-2 was smooth and similar to normal skin without scar formation. Furthermore, NC-GL and SCGL3-2 were capable of quickly promoting the growth of new rat hair at the regenerated skin.

#### 3.14.2. Histopathological and Immunohistochemical Analysis

##### Histopathological Analysis

**Figure 9:**
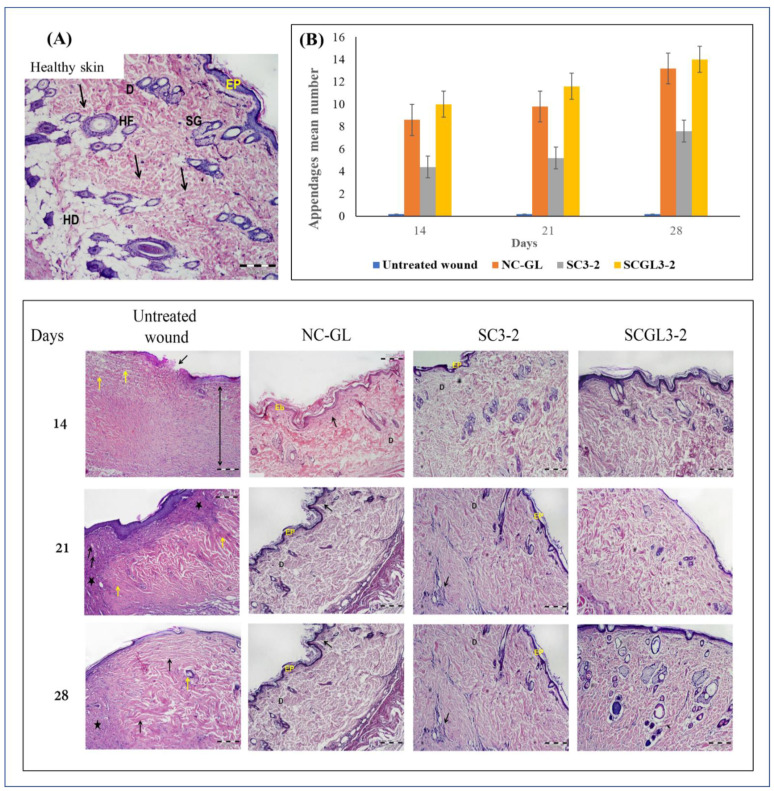
Histological evaluation of skin at days 7, 14 and 28 post-excision. (**A**) Histopathological micrographs of H&E-stained skin sections at days 7, 14 and 28 post-excision: The healthy thin skin shows a continuous epidermis (EP), loosely arranged collagen bundles (arrows) and plentiful hair follicles (HF). D: dermis, SG: sebaceous glands, HD: hypodermis. The untreated wound control at the 14th day shows a discontinuous epithelium (black arrow), excessive dermal granulation tissue with cellular infiltration (double head arrow) and parallel collagen bundles (yellow arrows). At day 21, the wound exhibits an intense inflammatory infiltrate (asterisks), congested capillaries (black arrows) and hyalinized collagen bundles (yellow arrows). After 28 days, the dermis shows parallel hyalinized collagen bundles (black arrows), persistence of areas of granulation tissue (asterisk) and few dermal appendages (yellow arrow). The NC-GL-treated wounds show complete bridging of the epidermis (EP), a gradual increase in properly arranged collagen and a gradual subsidence of a mild inflammatory infiltrate (arrows). The wounds treated by SC3-2 show a continuous epidermis (EP), organized collagen bundles within the dermis (D) and focal areas of cellular infiltration (arrows). The wounds treated by SCGL3-2 show apparently normal histological features. (H&E stain, Mic. Mag 100×) (**B**) Morphometric analysis of skin appendages. Statistical comparison between the studied groups is according to the mean number of skin appendages per microscopic field (*n* = 5).

**Figure 10:**
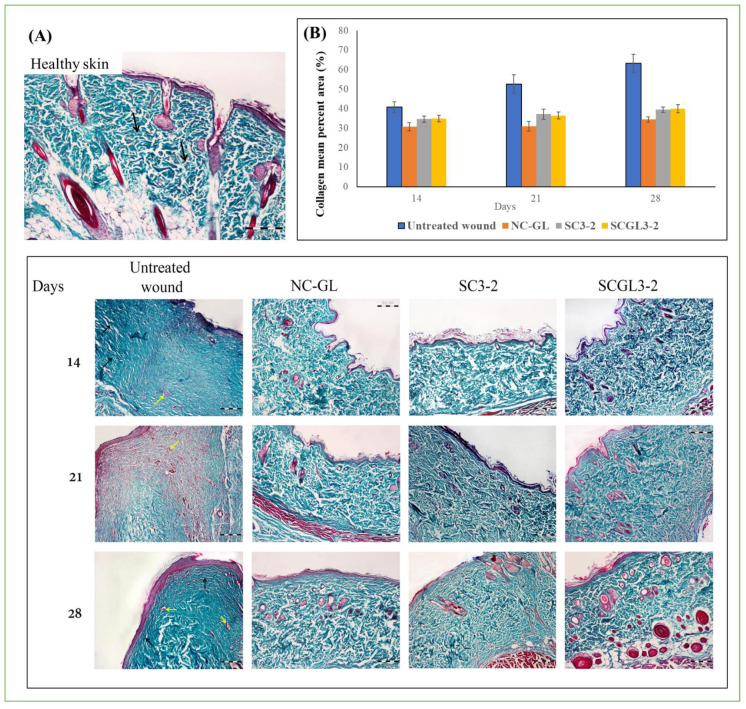
Masson’s trichrome staining of regenerated skin showing deposition of collagen fibers on days 14, 21 and 28: (**A**) Photomicrographs of healthy skin showing loosely arranged bundles of collagen (arrows). The untreated wounds appearing with parallel collagen bundles (black arrows), cellular infiltration and scattered congested vascular channels (yellow arrows). The wound treated by NC-GL showing deposition of fine, properly distributed collagen bundles. The wounds treated by SC3-2 showing apparently normal organization of collagen. The wound treated by SCGL3-2 showing collagen bundles, more or less similar to the normal control pattern. (Masson’s trichrome, Mic. Mag 100×) (**B**) Morphometric analysis of average percent area of collagen. The statistical comparison between the studied groups is according to the average percent area of collagen per microscopic field (*n* = 5).

Histological analysis of the regenerated skin on days 14, 21 and 28 post-excision was performed by H&E and Masson’s trichrome staining, as shown in Figure 9 and Figure 10.

##### H&E-Stained Sections

The healthy skin showed a continuous keratinized stratified squamous epithelium (epidermis). The underlying dermis revealed sparse collagen bundles arranged in a basket weave-like pattern, with numerous hair follicles and sebaceous glands (Figure 9A).

On the other hand, at 14 days post-wounding, the untreated wound group revealed discontinuity in the surface epithelium. The underlying dermis exhibited dense granulation tissue and parallel collagen fibers, together with a remarkable increase in the cellularity (Figure 9A). On day 21, the dermis appeared with an intense inflammatory infiltrate, congested capillaries and hyalinized thick collagen bundles. This revealed scar formation, as evidenced by the deposition of an abnormal excess amount of dermal collagen bundles that were arranged parallel to the skin surface. Additionally, the basement membrane of the epidermis that developed over the scar was flatter than the healthy skin, with no rete pegs that normally penetrate the dermis. In addition, the scar did not contain dermal appendages such as hair follicles and sebaceous glands [4]. After 28 days, no signs of improvement were detected (Figure 9A).

The enhancement in dermal and epidermal skin regeneration which is formed of well-structured epithelial layers with the absence of scarring crusting or intraepithelial inflammatory cells is indispensable for wound healing [103]. Herein, enhanced dermal and epidermal regeneration was achieved following treatment with NC-GL, SC3-2 and SCGL3-2. As for NC-GL-treated wounds, after 14, 21 and 28 days, they demonstrated complete re-epithelization of the epidermis, a gradual increase in loosely arranged collagen bundles, a gradual decrease in a limited undersurface inflammatory cellular infiltrate and a gradual increase in dermal appendages (Figure 9A). SC3-2-treated wounds showed focal areas of cellular infiltration that showed gradual regression over the studied period. There was a gradual increase in the amount of collagen bundles and skin appendages. Finally, examination of SCGL3-2-treated wounds revealed histological features similar to the healthy skin (Figure 9). The dermis appeared with randomly oriented and loosely arranged collagen bundles, together with multiple hair follicles and sebaceous glands.

Histomorphometric analysis of skin appendages is shown in Figure 9B. The results display a significantly lower number of dermal appendages in the untreated wound group in comparison to all three treated groups over the study period (*p* < 0.05). A pronounced enhancement in the recovery of skin appendages was attained by NC-GL and SCGL3-2, with no statistically significant difference between the two groups. On day 28, the number of appendages in the SCGL3-2-treated group was not significantly different (*p* > 0.05) from the healthy skin (14 ± 3 and 19 ± 4, respectively).

##### Masson’s Trichrome Stain

Collagen remodeling and maturation were evaluated by Masson’s trichrome staining. Collagen regeneration plays a crucial role in the wound healing process [104]. The normal skin showed loosely arranged short dermal bundles of collagen. In the untreated wound group, the collagen bundles were extensively deposited, thickened and mostly oriented parallel to the surface (Figure 10A). In the other three studied groups, collagen was properly sparsely arranged. It increased in amount gradually over the studied time points.

Morphometrically (Figure 10B), the collagen mean percent area in the untreated wound group was significantly increased in comparison to the healthy skin and all three treated groups. Over the three time intervals, NC-GL displayed a collagen percent area comparable to the healthy skin group. On the other hand, the SC3-2- and SCGL3-2-treated groups showed a slightly higher collagen percent area compared to NC-GL and the healthy skin group, with no statistically significant difference between the two scaffold-treated groups (*p* > 0.05).

Histological examination of the NC-GL-treated wounds demonstrated accelerated wound healing, and an early appearance of dermal appendages. This result correlates with that of several studies [26,105] that have proven the ability of GL to decrease the recovery time and increase the cellular proliferation of fibroblasts, keratinocytes and endothelial cells. Topical GL was shown to augment epithelialization and regulate granulation tissue formation, leading to the early beginning of the proliferative phase through the induction of the expression of numerous pro-healing factors such as IGF-1, TGF-β and IL-10 [24]. Additionally, the angiogenesis acceleration of GL was reported to be a key factor in proper wound healing as it is required for the adequate transport of nutrients, inflammatory cells, cytokines and chemokines to the wound site [10].

Despite the close similarity of skin macroscopical features observed following treatment with NC-GL and SCGL3-2, microscopical examination revealed an improvement in wound healing observed following NC-GL incorporation into the SC3-2 scaffold. The results of the CO/CS scaffold groups demonstrate improved wound healing and deposition of a significant amount of properly arranged collagen bundles, which is expected to provide more tensile strength to the wounded area. These results correlate with those of previous studies [11,16] showing the high cytocompatibility of CO/CS scaffolds, creating a suitable area for the proliferation of fibroblasts, thus leading to better secretion of more organized densely packed collagen fibers in the dermis [26]. Once the amount of collagen increases, the fibroblasts will be further promoted to fibrocytes, and thereby, the wound executes the healing process [11]. In the wound healing process, fibroblasts and capillaries must invade the clot to form a contractile granulation tissue that can draw the wound margins together [10]. Chitosan molecules also play a crucial role in healing by facilitating cell growth and proliferation as well as organizing the deposition of collagen [15,106]. Additionally, earlier observations showed that the combination of collagen and chitosan acts as a supportive scaffold for all growth factors producing cells, inducing faster tissue organization and regeneration [99,107].

##### Immunohistochemical Analysis

**Figure 11:**
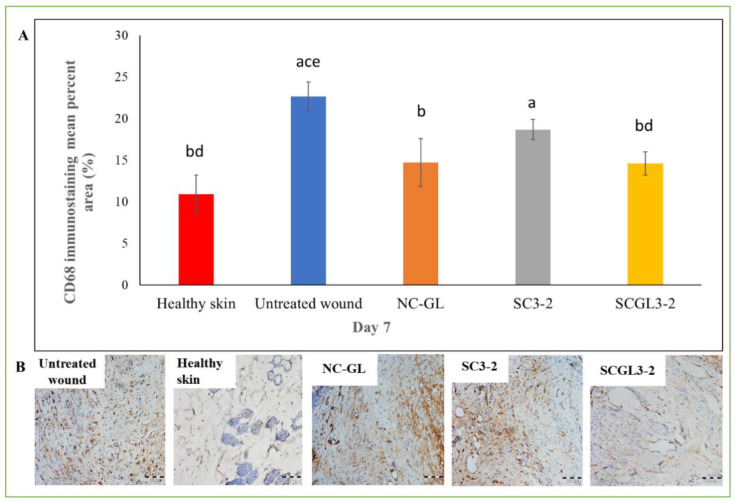
Immunohistochemical analysis of inflammatory reaction using CD68 staining: (**A**) Percentage of CD68 expression. The statistical comparison between the studied groups is based on the average percent area of CD68-positive immuno-reaction per microscopic field (*n* = 5). ^a^ *p* < 0.05 vs. healthy skin, ^b^ *p* < 0.05 vs. untreated control, ^c^ *p* < 0.05 vs. NC-GL, ^d^ *p* < 0.05 vs. SC3-2, ^e^ *p* < 0.05 vs. SCGL3-2. (**B**) Skin sections showing positive (brown) staining. (Immuno-stained sections Mic. Mag 200×).

CD68 is routinely used as a histochemical/cytochemical marker of inflammation associated with the involvement of monocytes/macrophages. As shown in Figure 11, seven days following wound induction, the untreated wound group exhibited a highly significant increase (*p* < 0.05) in CD68-positive macrophages in comparison with the healthy skin group. A slight reduction was observed following treatment with SC3-2 (22.63 ± 1.73 and 18.65 ± 1.2% for the untreated wound and SC3-2, respectively). A further reduction in CD68 percent area was observed for NC-GL and SCGL3-2 (14.73 ± 2.9 and 14.59 ± 1.4%, respectively), reaching a value that was slightly significantly different compared to the healthy skin group (10.92 ± 2.27%), indicating a decrease in inflammation in the wound area. The decrease in the inflammatory phase promotes granulation tissue formation and rapid cellular proliferation. This is explained by the well-reported anti-inflammatory effect of GL through the down-regulation of inflammatory cells [25], and the ability of the collagen/chitosan combination to inhibit bacterial growth and wound infection [108].

## 4. Conclusions

In the current study, glibenclamide was selected as a repurposed drug with a well-reported anti-inflammatory effect and potential for promoting wound healing. For efficient applicability to full-thickness wounds, the drug was incorporated into bioactive collagen/chitosan composite scaffolds. Due to its highly hydrophobic nature, the glibenclamide formulation as Kolliphor HS15-stabilized nanocrystals resulted in improved wettability and dissolution, allowing for its incorporation into the hydrophilic polymeric scaffold. The variation in the polymer content and the ratio between collagen and chitosan, in addition to the loading of glibenclamide nanocrystals, demonstrated a significant effect on the physicochemical properties including the porosity, swelling, water holding capacity, in vitro degradation, mechanical strength and in vitro drug release. Scaffolds with a total polymer content of 3% with a collagen/chitosan ratio of 2:1, showing the most favorable properties, were selected for further evaluation, where they showed both in vitro and in vivo wound healing properties attributed to the combined activity of the drug and the bioactive polymers used. Hence, this study presents a nanocomposite bioactive scaffold as an effective patient-friendly formulation for skin regeneration without scarring.

## Figures and Tables

**Table 1:** Glibenclamide nanocrystal codes, formulation parameters and colloidal properties.

Code	Stabilizer	Stabilizer Concentration % *w*/*v*	GL/Stabilizer Ratio	Probe Sonication Time (min)	PS ± SD	PDI ± SD
NC-GL1	PVP K30	0.1	1:20	15	823 ± 15	0.48 ± 0.01
NC-GL2	PVA	0.1	1:20	15	853 ± 34	0.63 ± 0.03
NC-GL3	SLS	0.1	1:20	15	831 ± 16	0.34 ± 0.07
NC-GL4	PEG 4000	0.1	1:20	15	449 ± 5	0.23 ± 0.04
NC-GL5	PLX 188	0.1	1:20	15	431 ± 4	0.44 ± 0.16
NC-GL6	K-HS15	0.1	1:20	15	305 ± 12	0.24 ± 0.11
NC-GL7	K-HS15	0.1	1:20	5	352 ± 2	0.29 ± 0.01
NC-GL8	K-HS15	0.2	1:20	5	339 ± 6	0.23 ± 0.02
NC-GL9	K-HS15	0.1	1:50	5	328 ± 3	0.29 ± 0.09

In all tested formulations, 25 mg GL was incorporated. Abbreviations: PS, particle size; PDI, polydispersity index; PLX188, poloxamer 188; PVP, polyvinyl pyrrolidone; SLS, sodium lauryl sulfate; PVA, polyvinyl alcohol; K-HS15, Kolliphor HS15; SD, standard deviation.

**Table 2:** Formulation code, composition, porosity, pore size and swelling index at 24 h of collagen/chitosan scaffolds.

Formulation Code	Collagen/Chitosan	Total Polymer Content (% *w*/*v*)	Porosity	Pore Size, μm	Swelling Index (24 h)
SC3-0.25	1:4	3	32 ± 5	-	928 ± 55
SC3-0.5	1:2	3	33 ± 2	-	1190 ± 42
SC3-1	1:1	3	69 ± 5	133 ± 19	1110 ± 33
SC3-2	2:1	3	68 ± 6	141 ± 40	1543 ± 56
SC6-0.5	1:2	6	37 ± 4	-	507 ± 136
SC6-1	1:1	6	62 ± 8	184 ± 49	1133 ± 467
SC6-2	2:1	6	68 ± 6	204 ± 34	1561 ± 68
SCGL3-1	1:1	3	91 ± 4	288 ± 39	936 ± 32
SCGL3-2	2:1	3	94 ± 4	221 ± 61	1095 ± 37
SCGL6-1	1:2	6	68 ± 11	257 ± 24	978 ± 112
SCGL6-2	2:1	6	55 ± 18	93 ± 13	1186 ± 50

Abbreviations: SC; blank scaffold, SCGL; NC-GL-loaded scaffold.

**Table 3:** Hemocompatibility evaluation: protein adsorption (mg/g), whole blood clotting and percentage hemolysis of selected scaffolds.

Code	Protein Adsorption (mg/g)	Whole Blood Clotting (OD)	% Hemolysis
SC3-2	9.58 ± 1.19	0.78 ± 0.09	3.15 ± 0.23
SC6-2	8.78 ± 0.11	0.35 ± 0.04	24.13 ± 2.28
SCGL3-2	16.87 ± 1.05	0.63 ± 0.17	0.22 ± 0.31
SCGL6-2	15.90 ± 1	0.20 ± 0.02	5.87 ± 0.76

## Supplementary Materials

The following are available online at https://www.mdpi.com/article/10.3390/pharmaceutics13091469/s1. Table S1. Drug release kinetics data.
