# Correction: Youssef et al. Glibenclamide Nanocrystal-Loaded Bioactive Polymeric Scaffolds for Skin Regeneration: In Vitro Characterization and Preclinical Evaluation. *Pharmaceutics* 2021, *13*, 1469

**DOI:** 10.3390/pharmaceutics15041156

**Published:** 2023-04-05

**Authors:** Julie R. Youssef, Nabila A. Boraie, Heba F. Ibrahim, Fatma A. Ismail, Riham M. El-Moslemany

**Affiliations:** 1 Department of Pharmaceutics, Faculty of Pharmacy, Alexandria University, Alexandria 21523, Egypt; 2 Department of Histology and Cell Biology, Faculty of Medicine, Alexandria University, Alexandria 21523, Egypt

**Figure Correction**

In the original publication [1], there was a mistake in the legend for Figure 9 “Histological evaluation of skin at **days 7, 14** and 28 post-excision”. And there was a mistake in Figure 9A, in NC-GL group the same image appears for days 21 and 28. The correct Figure 9 appears below. The authors state that the scientific conclusions are unaffected. This correction was approved by the Academic Editor. The original publication has also been updated.

## Figures and Tables

**Figure 9:**
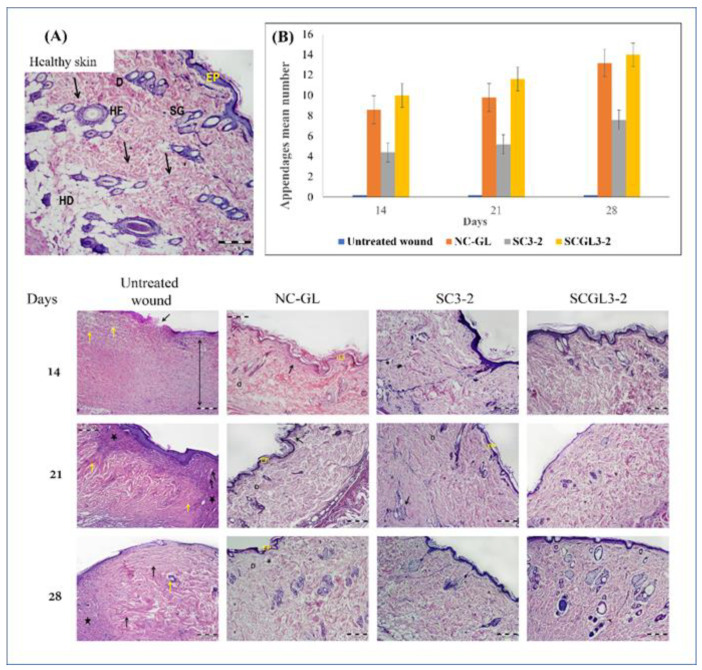
Histological evaluation of skin at days 14, 21 and 28 post-excision. (**A**) Histopathological micrographs of H&E-stained skin sections at days 14, 21 and 28 post-excision: The healthy thin skin shows a continuous epidermis (EP), loosely arranged collagen bundles (arrows) and plentiful hair follicles (HF). D: Dermis; SG: sebaceous glands; HD: hypodermis. The untreated wound control at the 14th day shows a discontinuous epithelium (black arrow), excessive dermal granulation tissue with cellular infiltration (double head arrow) and parallel collagen bundles (yellow arrows). At day 21, the wound exhibits an intense inflammatory infiltrate (asterisks), congested capillaries (black arrows) and hyalinized collagen bundles (yellow arrows). After 28 days, the dermis shows parallel hyalinized collagen bundles (black arrows), persistence of areas of granulation tissue (asterisk) and few dermal appendages (yellow arrow). The NC-GL-treated wounds show complete bridging of the epidermis (EP), a gradual increase in properly arranged collagen and a gradual subsidence of a mild inflammatory infiltrate (arrows). The wounds treated by SC3-2 show a continuous epidermis (EP), organized collagen bundles within the dermis (D) and focal areas of cellular infiltration (arrows). The wounds treated by SCGL3-2 show apparently normal histological features (H&E stain, Mic. Mag 100×). (**B**) Morphometric analysis of skin appendages. Statistical comparison between the studied groups is conducted according to the mean number of skin appendages per microscopic field (*n* = 5).
