# Supplementary material for: Glibenclamide Nanocrystal-Loaded Bioactive Polymeric Scaffolds for Skin Regeneration: In Vitro Characterization and Preclinical Evaluation
Source: Pharmaceutics. 2021 Sep 14;13(9):1469. doi: 10.3390/pharmaceutics13091469 (PMC8469322; doi:10.3390/pharmaceutics13091469)
Supplement: Supplementary file 1 [file pharmaceutics-13-01469-s001.zip › pharmaceutics-1351613-supplementary.pdf]

pharmaceutics

Supplementary Materials: Glibenclamide Nanocrystal-Loaded Bioactive Polymeric Scaffolds for Skin Regeneration: In Vitro Characterization and Preclinical Evaluation

Julie R. Youssef, Nabila A. Boraie, Heba Fathy Ibrahim, Fatma A. Ismail and Riham M. El-Moslemany

Table S1. Drug release kinetics data.

	**SC3-1**		**SC3-2**		**SC6-1**		**SC6-2**	
	**r**	**MSE**	**r**	**MSE**	**r**	**MSE**	**r**	**MSE**
First order	0.81	364.8	0.82	416	0.90	125.9	0.88	253
Higuchi	0.87	184.3	0.84	244	0.95	42	0.91	122.5
Korsmeyer-Peppas	0.95	40	0.93	77	0.97	16	0.96	44.5
Hixon-Crowell	0.78	419	0.78	493.6	0.89	139	0.85	303.8
Baker-Lonsdale	0.89	134.8	0.88	172.5	0.95	34	0.93	84
